# Enzymatic biosynthesis and immobilization of polyprotein verified at the single-molecule level

**DOI:** 10.1038/s41467-019-10696-x

**Published:** 2019-06-24

**Authors:** Yibing Deng, Tao Wu, Mengdi Wang, Shengchao Shi, Guodong Yuan, Xi Li, Hanchung Chong, Bin Wu, Peng Zheng

**Affiliations:** 1 State Key Laboratory of Coordination Chemistry, School of Chemistry and Chemical Engineering, Nanjing University, Nanjing, Jiangsu 210023 P. R. China; 2 School of Biological Sciences, Nanyang Technological University, 60 Nanyang Drive, Singapore, 637551 Singapore; 3 NTU Institute of Structural Biology, Nanyang Technological University, EMB 06-01, 59 Nanyang Drive, Singapore, 636921 Singapore

**Keywords:** Peptides, Atomic force microscopy, Single-molecule biophysics

## Abstract

The recent development of chemical and bio-conjugation techniques allows for the engineering of various protein polymers. However, most of the polymerization process is difficult to control. To meet this challenge, we develop an enzymatic procedure to build polyprotein using the combination of a strict protein ligase OaAEP1 (*Oldenlandia affinis* asparaginyl endopeptidases 1) and a protease TEV (tobacco etch virus). We firstly demonstrate the use of OaAEP1-alone to build a sequence-uncontrolled ubiquitin polyprotein and covalently immobilize the coupled protein on the surface. Then, we construct a poly-metalloprotein, rubredoxin, from the purified monomer. Lastly, we show the feasibility of synthesizing protein polymers with rationally-controlled sequences by the synergy of the ligase and protease, which are verified by protein unfolding using atomic force microscopy-based single-molecule force spectroscopy (AFM-SMFS). Thus, this study provides a strategy for polyprotein engineering and immobilization.

## Abstract (web summary)

Existing methods for protein polymer engineering suffer from low efficiency especially for synthesis large size polyproteins. Here, Deng et al. construct homo-polymer and co-polymer up to decamer by stepwise ligation and cleavage validated by atomic force microscopy-based single-molecule force spectroscopy.

## Introduction

Protein conjugation and polymerization is a natural biochemical process and has important applications in biomaterial and biomedicine engineering^1,2^. Compared with synthetic polymers, one of the unique features of most biopolymers, such as protein, is a uniform structure with a well-controlled sequence of amino acids, while multi-domain protein consists of similar or different protein subdomains. This clustering of the same or different protein domains often results in enhanced biological function and stability^3^. Several biochemical reaction-based methods, especially the cysteine-based coupling, have been developed for building protein polymer, in which protein monomer is designed with additional cysteines or specific residues as the basic unit for ligation^4,5^. However, unlike natural multi-domain proteins, it is rarely reported that these polymers and biomaterials are of well-controlled subunit sequence like their natural origin, and a bio-synthetic route for this purpose remains a key challenge. Another approach is to build the complete gene into one open reading frame for the artificial protein oligomer, just like the natural way. For example, a so-called polyprotein strategy has been developed to build protein oligomer to mimic natural modular protein^6–8^. The fused polyprotein comprises identical or different multiple protein domains whose genes are built using recombinant DNA technology. However, the engineering of toxic or large-sized protein polymer is often challenging. Also, many proteins such as metalloprotein and delicate enzyme may need purification as a monomer. Thus, the application of recombinant DNA technology for building polyprotein is also limited.

**Fig. 1:**
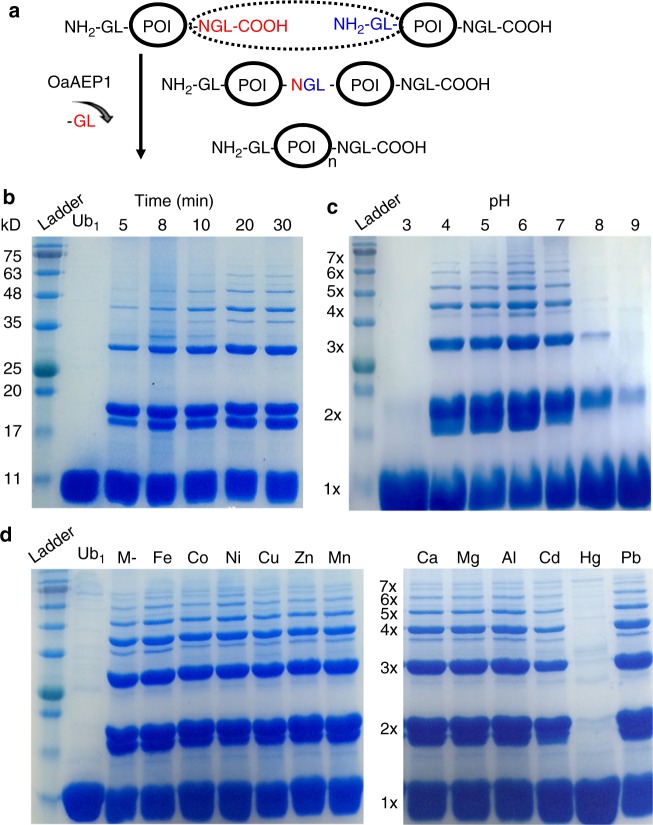
Protein polymerization by OaAEP1. **a** Principle of OaAEP1-alone for protein polymerization is based on a GL-POI-NGL protein unit in which an N-terminal NGL (colored in red) reacts with a C-terminal GL (colored in blue) and leads an NGL linkage. **b**–**d** The PAGE gel results of ubiquitin polymerization indicate the formation of a protein polymer mixture with a varied length under different conditions. The reaction is fast (completed in 20 min) with the formation of protein polymer at least up to a pentamer (**b**). The polymerization is robust under acidic pH from 4 to 7 (**c**), as well as in the presence of most biologically-relevant metal ions (**d**), except Hg

To address this challenge, we develop an enzymatic, stepwise construction of protein polymer/polyprotein with a relatively-precise controlled sequence using a protein ligase and a protein protease. OaAEP1 is a recently developed, efficient and strict endopeptidase, which links two peptides/proteins covalently as a peptide bond through two termini in less than 30 min^9^. It requires a ligation unit with only two N-terminal GL residues (NH_2_-Gly-Leu) and three C-terminal NGL residues (Asn-Gly-Leu-COOH) (Fig. 1a)^10,11^. Thus, based on the ligation unit GL-POI-NGL (POI: protein of interest), OaAEP1 can be used to build polyprotein with an uncontrolled sequence in the solution, similar to the bi-cysteine or other ligase-based coupling methods^4,12^. The synthesized polyprotein is characterized by the SDS-PAGE gel method, at the ensemble level. Moreover, the designed polymers are unambiguously verified by protein domain unfolding using AFM-based SMFS, at the single-molecule level. AFM-based SMFS can mechanically unfold each protein domain and characterize protein mechanics^13–23^. The unfolding of polyprotein leads to a characteristic sawtooth-like force-extension curve, in which each force peak corresponds to the unfolding of each protein domain^24–28^. Thus, the polymerization number of the protein polymer can be directly counted to verify our design^29–31^. A poly-ubiquitin molecule is built and characterized. Compared with the PAGE gel method, the protein is measured under a native condition at room temperature, and only well-folded protein shows expected stability. Also, any linker effect between ligated proteins can be examined. Consequently, SMFS measurement of the protein polymer not only confirms the polymer design but also provides complementary information about protein stability and folding^32–34^.

In addition to the polyprotein engineering, OaAEP1 is implemented for protein immobilization for SMFS, together with the specific cohesin-dockerin (Coh-XDoc) receptor-ligand pairs^35–37^. This configuration ensures the complete unfolding of all protein subdomains. And the polymerization number is counted correctly, Traditionally, the polyprotein sample is deposited on a glass coverslip and is picked up by AFM tip randomly through a non-specific interaction^6,7^. Although this set up is simple, the pick-up ratio of the high-quality single-molecule event is very low (<0.01%). Based on the development of surface (bio)chemistry methods for covalent anchoring of protein such as thiol chemistry, chloroalkane chemistry, isopeptide bond, click chemistry and specific and reversible receptor-ligand pairs for reversible linkage of sample such as streptavidin-biotin, cohesin-dockerin, and other antigen-antibody interactions, site-specific anchoring and probing configuration for AFM experiment has become possible and is becoming a standard method now^36,38–43^. Our method provides another alternative for such a purpose which can be adopted in all these similar AFM systems. Only two short peptide tags are needed for the ligation, which leads to simple protein preparation without further chemical modification.

Moreover, this OaAEP1 ligase-mediate method is cysteine-free and achieved at the monomeric protein module level. It enables the study of the challenging protein system such as metalloprotein, which needs initial purification as a monomer. Because the overexpression of metalloprotein often results in different metal forms as a mixture, it requires additional purification to obtain a pure-metal form monomer for ligation^44,45^.

Lastly, we take advantage of a removable TEV protease site, which is compatible with OaAEP1 ligation, to achieve the stepwise protein polymerization on the surface. When a TEV site (ENLYFQ/G) plus a leucine (L) is engineered at the N-terminus of the protein unit as ENLYFQ/G-L-POI, the TEV cleavage results in an N-terminal GL residue as GL-POI, and is compatible with further OaAEP1 ligation. Consequently, an enzymatic, stepwise biosynthesis of polyprotein with a relatively well-controlled sequence is achieved. Thus, our enzymatic method provides a method for polyprotein sample preparation, both sequence-uncontrolled and controlled, as well as protein immobilization for single-molecule studies, especially for the complex metalloprotein system^46–50^.

## Results

### A sequence-uncontrolled polyprotein built by OaAEP1-only

Protein ubiquitin (Ub), which has been well characterized by single-molecule AFM before, was chosen for demonstration. The poly-ubiquitin is also a natural signal for protein degradation with biological function. A construct GL-Ub-NGL was built for the OaAEP1 polymerization and reacted in the buffer solution. The Coomassie-stained SDS-PAGE gel results of the product clearly showed the rapid construction of Ub polymer at least up to a pentamer within 20 min (Fig. 1b). Quantitatively, ~25% of the protein unit was ligated to a dimer, 10% to a trimer, while ~60% of the protein remained a monomer (Fig. 1b). As expected, the polyprotein length was uncontrolled, and the yields diminished rapidly as the chain grew. Nevertheless, its yields and dispersity were comparable to most other protein monomer-based polymerization method, such as the bi-cysteine and sortase-based method.

In addition, we found that OaAEP1 is also a robust ligase under harsh conditions, such as under the acidic solutions and in the presence of metal ions. First, the same ubiquitin polymerization reaction was tested under different pH levels. The SDS-PAGE gel results showed the polymerization was well-performed under acidic condition (pH 4 to 7) (Fig. 1c). Moreover, the reaction was performed in the presence of the most biologically-relevant metal ions. Twelve different metal ions including Fe(III), Co(II), Ni(II), Cu(II), Zn(II), Mn(II), Ca(II), Mg(II), Al(III), Cd(II), Hg(II), and Pb(II) were tested, respectively. The SDS-PAGE gel results showed that the reaction was not affected by most metal ions at the concentrations of 0.2 mM, except 0.002 mM for Hg (Fig. 1d, Supplementary Fig. 1). These concentrations are much higher than the free metal level in most metalloprotein solutions. These experiments indicate that OaAEP1 ligase is a versatile enzyme that is suitable for constructing challenging protein under harsh conditions.

**Fig. 2:**
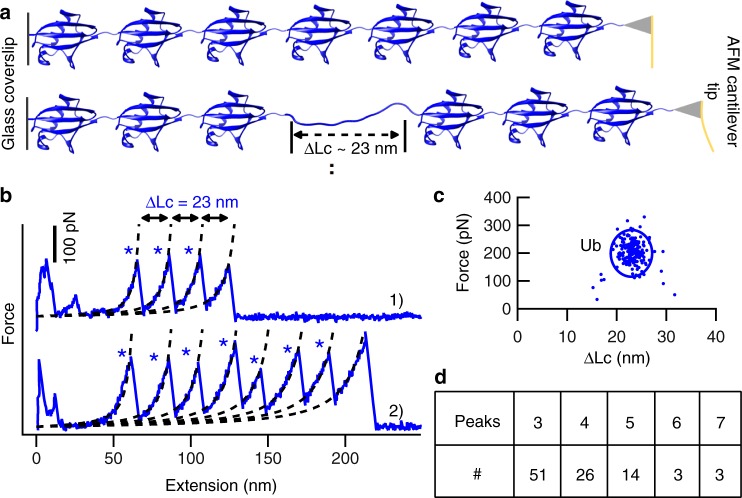
The characterization of OaAEP1-built polyprotein by AFM-based SMFS. **a** The schematic displays the unfolding of ubiquitin polyprotein by single-molecule AFM, which leads to an unfolding peak with ΔLc of ~23 nm. **b** Two representative force-extension curves of (Ub)_n_ are shown with expected ΔLc and different peaks indicated by blue star, three peaks in curve 1, and seven peaks in curve 2. **c** The scatter plot of the (Ub)_n_ unfolding experiment showed the relationship between protein unfolding force (202 ± 44 pN, average ± s.d., *n* = 198) and ΔLc (23 ± 2 nm, average ± s.d.). **d** The statistical analysis of the (Ub)_n_ indicated the number of curves with specific unfolding peaks. Most curves showed three (52%) and four (27%) unfolding peaks. Source data of Fig. 2c are provided as a Source Data file

Next, the polyprotein sample obtained by OaAEP1-only ligation was used directly for single-molecule AFM characterization and study. The sample purified by gel-filtration chromatography with higher polymerization degrees was used for better performance, in which Ub tetramer presented most (Supplementary Fig. 2). The protein solution was deposited on a clean glass coverslip and was then pressed and captured by AFM tip (Fig. 2a). Stretching the polyproteins resulted in a typical saw-tooth like force-extension curve with multiple peaks, which corresponded to the unfolding of each ubiquitin monomer (Fig. 2b). For example, seven unfolding peaks were observed in curve 2 of Fig. 2b, indicating that seven ubiquitin units were unfolded. Two key experimental results, the contour length increment (ΔLc) and the force (F) upon unfolding were analyzed, and the results are shown in Fig. 2c. The contour length increment is related to how many proteins residues are unfolded and extended upon protein unfolding. Previous single-molecule AFM unfolding experiments of ubiquitin polyprotein built by the recombinant DNA method showed a ΔLc of ~24 nm, which was from the full extension of 76 amino acids of Ub (76aa*0.36nm-4 nm = 23 nm, 4 nm is the distance between the N and C termini of the folded Ub), and an average unfolding force of 203 pN^51,52^. Here, our ligase-built ubiquitin polymer showed comparable results, with an average ΔLc and standard deviation of 23.1 ± 2 nm, and an average unfolding force and standard deviation of 202 ± 44 pN, number (*n*) = 198. These results validate our method for building polyprotein for single-molecule measurement. Moreover, it indicates no linker effect from the three new connection NGL residues between protein monomers as the same unfolding forces for Ub measured in our construct. Finally, there was a large dispersity, as many different numbers of ubiquitin unfolding peaks from one to seven were recorded for each molecule. Thus, more experiments were performed to obtain statistics. As shown in the Fig. 2d, most curves showed three Ub unfolding peaks (*n* = 51, 52%) and four Ub unfolding peaks (*n* = 26, 27%). The curves with only two or one ubiquitin molecules were not analyzed, as their signals were strongly affected by the non-specific interaction for a short molecule.

It is noted that Ub trimer (52%) was detected most instead of tetramer (27%), which was different from the previous polyprotein mixture identification (Supplementary Fig. 2). Here, the polyprotein sample absorbed on the coverslip was picked up at a random location along the long protein polymer, and by a weak non-specific interaction between the protein and AFM tip or coverslip. It is possible that the full-length protein polymer may not have been completely captured and unfolded. For example, the seven unfolding peaks of Ub observed above (Fig. 2b) cannot guarantee that a ubiquitin heptamer was captured. It was perhaps an octamer, but the tip only pressed on the seventh Ub domain, and one domain was not unfolded and counted. Another possibility is that an octamer was fully captured, while the stability of one domain was higher than the non-specific protein detachment force from either the AFM tip or the glass coverslip. Thus, the domain remained folded when the octamer was detached. Both scenarios have led to force-extension curves detected with fewer unfolding peaks and a large dispersity.

### The covalent immobilization of (poly)protein in the AFM system

**Fig. 3:**
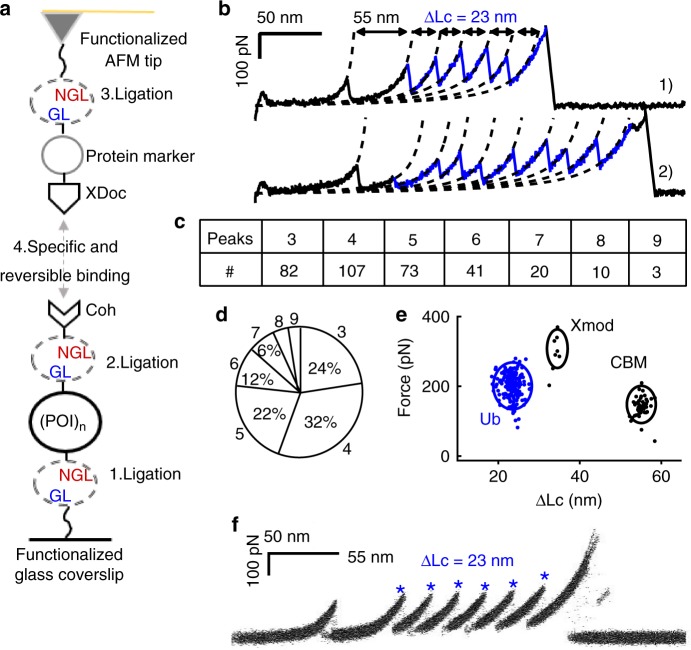
The OaAEP1-dependent polyprotein immobilization verified by single-molecule AFM. **a** The schematic shows that OaAEP1 facilitates a covalent anchoring of (poly)protein on a glass surface as well as a specifically probing by an XDoc-functionalized AFM tip. **b** Representative curves of (Ub)_n_ in the specific attachment showed a high detachment force from the Coh-XDoc interaction and more unfolding peaks. **c**, **d** The statistical analysis of the (Ub)_n_ indicated the number of curves with a specific number of ubiquitin peaks. Most curves showed the number of Ub unfolding peaks between three and six (>90%). **e** The scatter plot shows the relationship between unfolding/rupture force and ΔLc for (Ub)_n_, Xmodule of the XDoc (ΔLc = 35 nm), and the CBM. **f** The overlay of all curves with CBM unfolding first show the complete unfolding of the polyprotein (Ub)_6_ using the OaAEP1-dependent polyprotein immobilization method (*n* = 52). The covalent immobilization with a high detachment force enables the complete unfolding of all subdomains in the polyprotein. Source data of Fig. 3e are provided as a Source Data file

To solve this problem, a covalent attachment of the protein sample in the AFM system with defined immobilization geometry and strong attachment force was developed using OaAEP1 as well. We demonstrated this application based on a strong and reversible type III cohesin-dockerin-Xmodule (Coh-XDoc, or Coh-Doc) receptor-ligand pair developed by Dr. Nash and Dr. Gaub for single-molecule studies^35,53^. Protein polymer, GL-(POI)_n_-NGL, as obtained above, was directly used here. First, it was covalently linked to the NH_2_-Gly-Leu functionalized glass coverslip using OaAEP1 through its C-terminal Asn-Gly-Leu residues. Then, it was ligated with the Coh-NGL through its N-terminus as Coh-(POI)_n_-NGL-Glass. Finally, it was directly probed by a Protein Marker-XDoc functionalized AFM tip forming a complete force loop: AFM Tip-Protein Marker-(XDoc-Coh)-(POI)_n_-Glass (Fig. 3a). The experimental details are described in Supplementary Methods. (Ub)_n_ obtained previously was used directly and measured in this configuration. It showed similar unfolding force and ΔLc results to before (Fig. 3b). In contrast, from the statistical analysis, it is clear that protein with a larger polymerization number was detected with higher frequency, and the tetramer presents most now (32%, Fig. 3c, d). This indicates the polyprotein was stretched between the two ends in this configuration. Fifty-two curves were randomly selected for force analysis (Fig. 3e). The force peak with a ΔLc of ~55 nm was from the unfolding of the protein marker CBM (cellulose-binding module), and sometimes Xmodule in the XDoc unfolded before the Coh-XDoc complex dissociated, with a ΔLc of ~34 nm ^35^.

To verify all subdomains were unfolded in this covalent attachment setup, a (Ub)_6_ polyprotein with a known number of subdomains was built by the recombinant DNA method and tested. Coh-(Ub)_6_ was used for coverslip attachment, and a CBM-XDoc was used for the tip functionalization. As a result, a polyprotein CBM-(XDoc-Coh)-(Ub)_6_ including one marker protein CBM and six ubiquitin was used. Most AFM measurement showed an expected full-length polyprotein unfolding scenario with six Ub (292 out of 322, 91%), as demonstrated by the overlap of the force-extension curves with CBM unfolding first. (Fig. 3f, *n* = 52). Consequently, this OaAEP1-facilitated covalent protein immobilization method enables the complete unfolding and correct counting of folded subdomains in the polyprotein molecule.

### Pure-metal form polyprotein built from the purified monomer

**Fig. 4:**
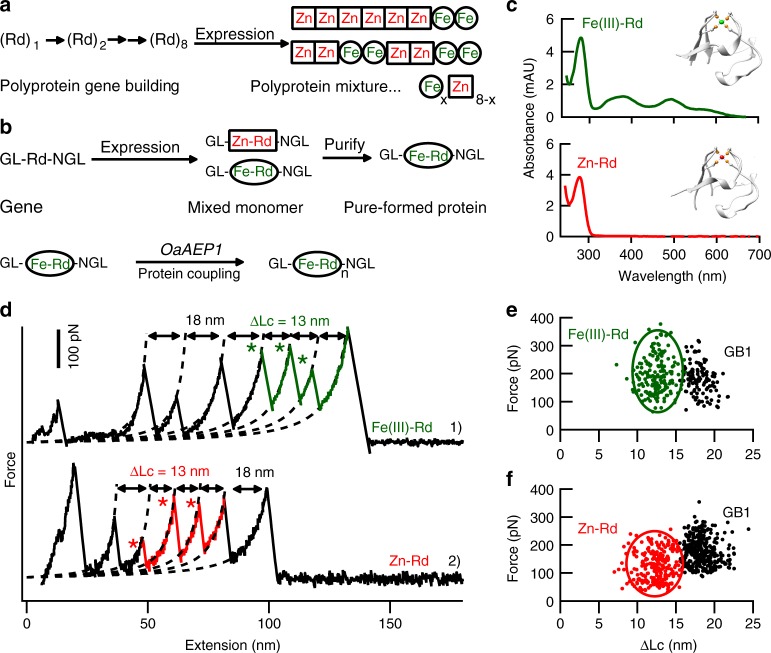
The construction of polyprotein with pure metal form by OaAEP1. **a** The schematic shows that the recombinant DNA method-based polyprotein construction strategy results in a polyprotein (Rd)_8_ with protein domains of different and uncontrolled content (metal form). **b** By expressing the protein as a monomer, it can be purified first, and then be ligated as a pure metal-form polyprotein. **c** The UV-Vis absorbance spectra of purified rubredoxin monomers, GL--GB1-Fe(III)-Rd-NGL (Top spectrum, colored in green, PDB code for Fe(III)-Rd:1BRF) and GL--GB1-(Zn)-Rd-NGL (Bottom spectrum, colored in red, PDB code for Zn-Rd: 1IRN). The Fe(III)-form Rd showed a characteristic UV-Vis at the wavelength of 495 nm and 579 nm. **d**–**f** Pure metal form GL-(GB1-Rd)_n_-NGL, Fe(**e**) and Zn(**f**) built by OaAEP1 showed comparable AFM results as the bi-cysteine built polyprotein. The ΔLc value of ~13 nm is observed as expected for rubredoxin unfolding. Source data of Fig. 4c, e and f are provided as a Source Data file

Our monomer-based ligation method enables the study of more challenging protein system such as metalloprotein, which sometimes shows several different metal-forms and needs purification as a monomer first. A well-characterized iron-sulfur protein rubredoxin (Rd) with a native Fe(Cys)_4_ metal center was chosen for demonstration. The overexpression of rubredoxin in *E. coli* results in a mixture of native iron-form and zinc-substituted form, whose polyprotein built by the classic recombinant DNA method lead to a mixed and uncontrolled metal form (Fig. 4a)^44^. Instead as a monomer, the protein mixture solution can be separated into pure Fe-Rd and pure Zn-Rd firstly using ion-exchange chromatography (Fig. 4b). The purity was confirmed by their characteristic UV-Vis spectra, respectively (Fig. 4c). As a result, the pure-metal form Rd can be polymerized to poly-metalloprotein sample (Fe-Rd)_*n*_ and (Zn-Rd)_*n*_ for AFM measurement. Here, we fused the marker protein GB1 to Rd as GL-(GB1-Rd)-NGL for the experiment (Fig. 4d). The protein GB1 was used here as a single-molecule fingerprint with known ΔLc of 18 nm as well as an internal force caliper (178 pN). Previous cysteine-based protein coupling method using C-(GB1-Rd)-C showed that the unfolding force of Fe(III)-Rd was 211pN with ΔLc of 12.6 nm^54^. Here, the AFM measurement of the two polyproteins built by our method (Fig. 4e) showed comparable results: 194 ± 63 pN (*n* = 184) and 12.6 ± 1.5 nm for Fe(III)-Rd (Fig. 4e), and 124 ± 52 pN (*n* = 246) and 12.4 ± 1.7 nm for Zn-Rd (Fig. 4f).

### Sequence-controlled polyproteins built on the glass surface

**Fig. 5:**
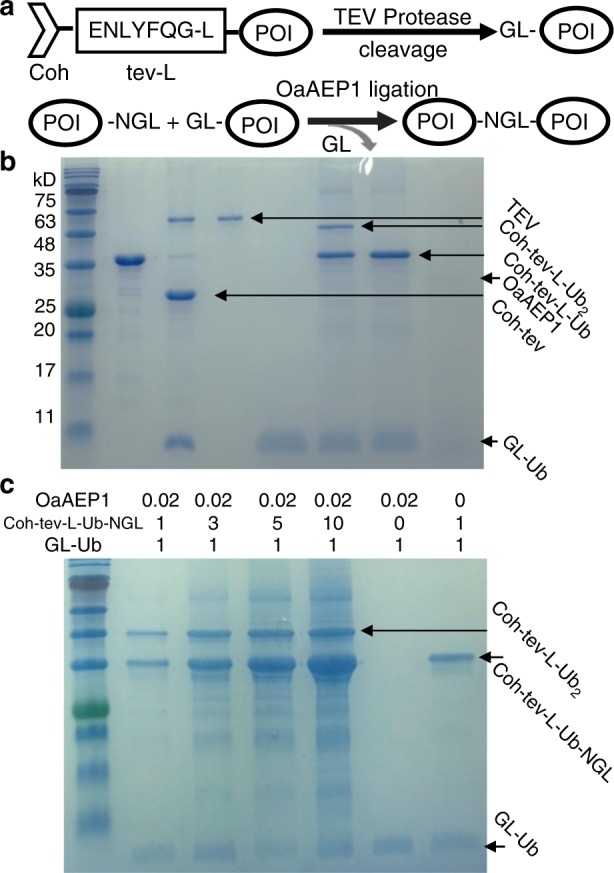
The high-efficiency construction of a protein dimer using OaAEP1 and TEV protease. **a** The schematic shows a removable N-terminal TEV site functions as a protective unit and its cleavage leads to a stepwise ligation using OaAEP1. **b** SDS-PAGE gel results showed the cleaved GL-Ub from Coh-tev-L-Ub reacted well with Coh-tev-L-Ub-NGL forming a Ub dimer as Coh-tev-L-(Ub)_2_. Lanes 1–4 show Coh-tev-L-Ub, the protein mixture after TEV cleavage, pure TEV protease (sfGFP-TEV) and purified cleaved GL-Ub. Lanes 5–7 show the ligation product mixture using the cleaved GL-Ub and Coh-tev-L-Ub-NGL for ligation, the reactant mixture without OaAEP1 is used as a control and pure OaAEP1. **c** The OaAEP1 ligation efficiency is proportional to the ratio between the two reactants, Coh-tev-L-Ub-NGL and GL-Ub. The efficiency increases from 20% when the ratio is 1 to 1, to 40% when the ratio is 3 to 1, to 50% when the ratio is 5 to 1, and reached 75% at the ratio of 10 to 1. The yield was calculated based on the band intensity of the dimer product Coh-tev-L-Ub_2_ using the software image J

To rationally control the sequence of the polyprotein built by OaAEP1, we first validated in principle that the TEV cleavage site is compatible with our OaAEP1 ligation system for a stepwise protein polymerization. As shown in the schematic of Fig. 5a, when a TEV site (ENLYFQ/G) plus a leucine (L) was added at the N-terminal part of a protein, the TEV protease cleavage results in an N-terminal Gly-Leu. As a result, the cleaved protein was then compatible with further OaAEP1 ligation and ultimately led to a protein polymer. Ubiquitin was used here for demonstration again. The SDS-PAGE gel result proved the validity of such a procedure for the construction of ubiquitin dimer based on Coh-tev-L-Ub (for cleavage) and Coh-tev-L-Ub-NGL (for ligation) (Fig. 5b).

However, the ligation efficiency for OaAEP1 was not sufficiently high enough for efficient polymerization. Previously, the OaAEP1-only ligation resulted in Ub dimer formation with a yield of 25%. Here, for a one-step ligation, the efficiency was of 20%. Although this is satisfactory for the one-step protein labeling or a dimer construction, it is still challenging to build a relatively long protein oligomer/polymer. The yield decreases exponentially after several rounds of reaction. Consequently, it is necessary to increase ligation efficiency.

For a chemical reaction, when one reactant is added in excess, the chemical equilibrium will be pushed toward the product. Thus, we modified the ratio between the two reactants to increase the yield. The one-step ligation between Coh-tev-L-Ub-NGL and GL-Ub was used here for the test. When the two reactants were in an equal molar ratio, the ligation efficiency was 20%. By increasing their ratio from 1 to 3, the yield improved to 40%, and 50% at a ratio of 5. Finally, when a ratio of 10 to 1 was used, the efficiency increased to 75% (Fig. 5c). Here, the ligation efficiency is calculated based on the formation of the product, Coh-tev-L-(Ub)_2_ dimer, whose band color in the gel intensifies clearly as the reactant ratio increases. To resolve between the reactant monomer Coh-tev-L-Ub-NGL and the product dimer, the sample migrates for a long time. As a result, the band of the other smaller reactant monomer GL-Ub is unclear upon the long migration. By increasing the concentration ten times for all the samples, the decrease of this monomer can also be detected and used for the efficiency calculation with similar results (Supplementary Fig. 3). Consequently, the yield (75%) for OaAEP1-ligation is obtained and is enough to build a polyprotein.

**Fig. 6:**
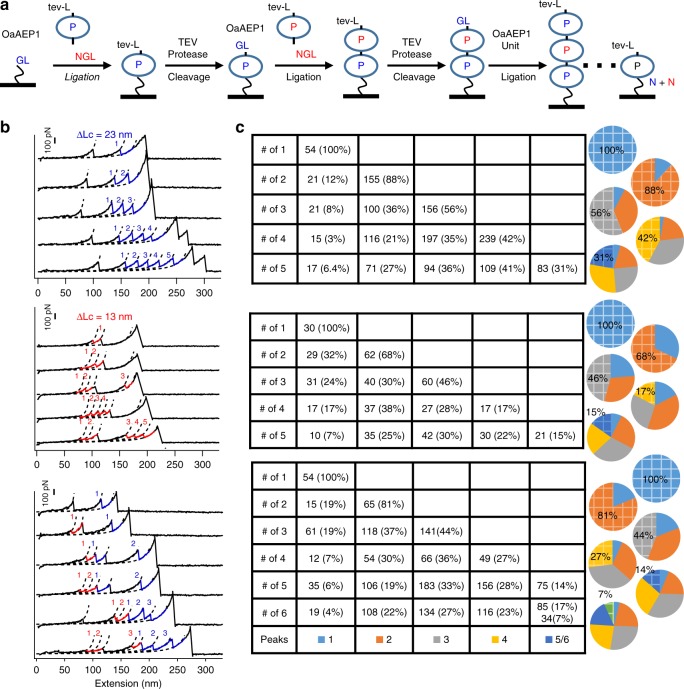
Stepwise construction of polyproteins with a rationally-controlled sequence. **a** The schematic of the stepwise, ligation and cleavage procedure to construct sequence-controlled polyprotein on the glass surface. **b** Representative curves with maximum unfolding peaks from the unfolding experiments of homo-polyprotein (Ub)_5_, top graph, (Rd)_5_, middle graph, and (Ub-Rd)_3,_ bottom graph, are shown, which were detected during each polymerization step from one to five/six for the same sample. **c** The statistical analysis of the number of the curve with specific unfolding peaks, corresponding to the left curves

We then built the protein polymer on a functionalized glass surface based on the stepwise OaAEP1 ligation and TEV cleavage reaction (Fig. 6a). First, the C-terminus of the protein unit was linked to the glass surface which naturally protects the protein unit from the sequence-uncontrolled polymerization. In addition, it allows the removal of excessive protein monomer and enzymes by simple buffer washing. Moreover, the resultant polymer was ready for single-molecule AFM characterization. We first ligated the monomer Coh-tev-L-Ub-NGL on the NH_2_-Gly-Leu (GL) functionalized glass coverslip as the Coh-tev-L-(Ub)_1_-NGL-Glass, denoted as (Ub)_1_ here for simplicity. The cohesin incorporated here was for single-molecule AFM measurement using an XDoc-functionalized tip. The AFM results of (Ub)_1_ showed the corresponding unfolding event with only one Ub peak (Number, *N* = 54). Then, (Ub)_1_ was cleaved by protease TEV as GL-(Ub)_1_-NGL-Glass to expose the GL residues for the second-round ligation. By adding the protein unit Coh-tev-L-Ub-NGL in excess with OaAEP1, Coh-tev-L-(Ub)_2_-NGL-Glass as a ubiquitin dimer was obtained. (Ub)_2_ was also characterized by single-molecule AFM, showing the corresponding dimer formation (*N* = 155, 88%). By repeating this stepwise cleavage and ligation procedure, we obtained ubiquitin polymer up to (Ub)_5_. The AFM experiments showed that 31% (*N* = 83) of curves had five Ub unfolding peaks (Fig. 6c). Through the whole process, the same polyprotein sample was characterized at each polymerization stage using the same AFM tip, and the maximum unfolding peaks of polymer picked up corresponded to the desired length (Fig. 6b). Nevertheless, there was still a fraction of molecules that were not reacted, as the ligation efficiency was not 100%. Indeed, when the polymer grew to hexamer, the yield became even lower (Supplementary Fig. 4).

Similarly, metalloprotein Rd was constructed as a sequence-controlled polyprotein on the glass surface and characterized. It demonstrated the feasibility of this stepwise method for constructing metalloprotein polymer (Fig. 6b). Finally, the protein copolymer (Ub-Rd)_3_ was also built by adding the protein units Ub and Rd one by one. Qualitatively, protein polymer built up to decamer can still be captured (Supplementary Fig. 5). Nevertheless, the yield becomes too low after five ligation cycles, and only a few curves can be found. Generally speaking, a protein pentamer can be obtained with a reasonable yield.

## Discussion

In this study, we have developed a simple, enzymatic methodology for building both sequence-uncontrolled and sequence-controlled polyproteins using protein ligase OaAEP1 and protease TEV. The protein ligation and polymerization were achieved under a mild condition, which should be applicable for most delicate proteins. In addition, the OaAEP1 is robust under harsh conditions, such as acidic solution and additional metal ions, which further expands the application of this method to build complex proteins. Most importantly, only two and three short residues as peptide tags are needed for the OaAEP1 ligation, which leads to minimal perturbance to the protein. The resultant peptide linkage between proteins is both thermally and mechanically stable, proved by the joint SDS-PAGE gel and single-molecule force spectroscopy experiments.

By adding one reactant in excess, the ligation efficiency increases from 20 to 75%. This allows for the construction of a relatively long protein oligomer. Statistically, pentameric protein, such as (Ub)_5_, can be obtained on the glass surface with a small polydispersity and percentage of ~30%. And a long protein decamer (Ub-Rd)_5_ can be obtained but only with several molecules under current ligation efficiency. Further improvement of the ligation efficiency or a new method to purify the resultant protein is necessary to achieve such a long protein polymer. Nevertheless, these results are already an advance compared with other ligase-based protein ligation method. Previous protein ligase-dependent polymerization seldom reported the construction of a protein pentamer, and the yield for a protein tetramer was <1% at the single-molecule level^12,55^. Thus, our method provides a tool for biotechnology and protein engineering, which is suitable for both protein coupling and immobilization.

There are other enzymatic methods which are well-suited for linking protein as a dimer or for protein labeling, such as sortase, split intein, bultease, and SpyCather-SpyTag^12,55–57^. Sortase is another similar peptidase which is promising for building long protein polymer. Several groups have used it for single-molecule AFM studies, both for protein covalent attachment and polyprotein construction, with important discoveries^12,53^. Unfortunately, the wild-type sortase is not a strict ligase and can hydrolyze the ligated linker itself. The engineering for a better sortase is under intense study with substantial improvements being developed^58^. The SpoonTag/SpoonCatcher system based on the SpyCather-SpyTag system is another powerful ligation method with a high ligation efficiency of >95% by forming an intermolecular isopeptide formation^59,60^. Thus, a long protein polymer like a decamer can be obtained in a similar, stepwise fashion with a high yield. By comparison, our OaAEP1 requires two and three additional amino acids at the two ends, and a short three amino-acid length linkage is present after ligation. Thus, either of these two methods may be applied based on different requirements.

From the perspective of single-molecule study, our method also provides a way to construct the polyprotein sample with a better-controlled length using monomeric protein unit. Recombinant DNA technology was employed to build polyprotein for single-molecule AFM study, which composes of identical or different multiple protein domains. Thus, it results in characteristic sawtooth like force-extension curves from the stepwise unfolding of each domain under force. This strategy significantly increases the data reliability and collection efficiency and has become the gold standard for AFM-based SMFS^7^. However, it relies on repetitive cloning cycles and is time-consuming. Recently, a much more efficient Gibson assembly-based method was developed for building long polyprotein genes^61,62^. Nevertheless, the engineering of toxic or large-sized protein polymer is often challenging, as the misfolding or the inability to express a large-size protein often occurs, and limits its application.

Another simple approach is to build the polymer at the monomeric protein level, by expressing individual protein monomers first and then conjugating them as a polyprotein. A bi-cysteine based protein coupling method was first developed and is widely used. By engineering two cysteines on a single protein, the formation of the intermolecular disulfide bond enables the construction of polyprotein and has led to many important discoveries^4,63^. This approach is efficient and enables the construction of larger protein like poly-GFP. A modified method was later developed consisting of a reducing-resist thiol-ether bond formation using maleimide-thiol chemistry^64^. Recently, similar ligase-based polymerization methods using sortase were also developed^12,55^. All these methods enable the pre-purification of protein monomer before polymerization. Thus, they are suitable for building complex protein such as metalloprotein. However, these polymerization methods all suffer from a sequence-uncontrolled protein polymer with a large dispersity. The protein unit is also linked in a mixed geometry of head-to-tail and head-to-head. For the sortase-based method, a long protein polymer was difficult to obtain due to the low ligation efficiency^12,55^. Here, our method utilizes a cysteine-free ligase OaAEP1 for protein ligation, polymerization, and immobilization with relatively high yield.

In conclusion, we develop an enzymatic method to synthesize both sequence-controlled and non-controlled polymerized protein. The robustness and high efficiency of the ligase enable the engineering and study of a wide range of protein, both delicate and complex, as well as providing an efficient way for both protein sample coupling and immobilization for single-molecule studies.

## Methods

### Protein engineering

The gene coding for protein of interest: ubiquitin from human (Ub), rubredoxins (Rd, Rd represents Zn-formed rubredoxin from *Clostridium pasteurianum*, if not specified, Fe-form rubredoxin from *Pyrococcus furiosus*), the B1 domain of immunoglobulin G (GB1), the cellulose-binding module (CBM), type III cohesin-dockerin-X module domain complex from *Ruminococcus flavefaciens* (Coh-Xmodule-Doc, or Coh-XDoc), *tobacco etch virus* (TEV) protease (fused with superfold GFP as GFP-TEV for use), elastin-like polypeptides (ELP) were ordered from Genscript Inc, respectively. Regular PCR procedure was used for the further addition of N-terminal GL and C-terminal NGL to the protein if needed. Typically, a three-restriction digestion enzyme system *BamH*I-*Bgl*II-*Kpn*I was used for connecting the gene of different protein fragments^7^. The same overhang after *BamH*I and *Bgl*II digestion allows the stepwise ligation between their fragments. All genes were finally confirmed by DNA sequencing from GenScript Inc. Typical protein overexpression and purification procedure, the general method for OaAEP1-only polymerization, and corresponding amino acid sequences are all provided in Supplementary Methods and Notes.

First, the pQE80L-POI or pET28a-POI expression plasmids were transformed into *E. coli* BL21(DE3) cells. Single colonies were picked into LB medium containing 100 µg mL^−1^ ampicillin sodium salt or 50 µg mL^−1^ kanamycin (continuous shaking, 37 °C, and 16–20 h). After grown to saturation, overnight cultures were diluted 1:50 into fresh LB media containing ampicillin sodium salt or kanamycin (continuous shaking, 37 °C, t ~3 h, except Fe(III)-Rd, and Zn-Rd which were overexpressed in M9 medium (M9 media supplemented with 0.4% glucose, 0.1 mM CaCl_2_, 2 mM MgSO_4_) with continuous shaking 6 h), and induced with 1 mM isopropyl β-D-thiogalactoside (IPTG) based on each protein when OD_600_ is ~0.6 (For rubredoxin, 1 mM FeCl_3_ or ZnCl_2_ is added). The bacterial cultures were allowed to incubate for an additional 4–6 h (37 °C). Finally, 400 mL bacterial culture was pelleted by centrifugation (13,260 × *g*, 25 min, 4 °C) and stored at −80 °C before purification.

The cells were then resuspended in 25 mL lysis buffer (50 mM Tris, pH 7.4) and lysed on ice using a Biosafer sonicator (15% amplitude for 30 min). The lysate was centrifuged (19,632 × *g*, 40 min, 4 °C) to pellet cell fragments and the supernatant fluids were applied to a Co-NTA or Ni-NTA affinity column (TALON) and washed with buffer containing 20 mM Tris, 400 mM NaCl, 2 mM imidazole, pH 7.4. The bound protein was eluted with elution buffer (20 mM Tris, 400 mM NaCl, 250 mM imidazole, pH 7.4). For rubredoxin we used an anion exchange chromatography (Mono Q 5/50 GL GE Healthcare) using a continuous salt gradient of 0–30% of buffer B (50 mM Tris, 1 M NaCl, pH 8.5) and then a size-exclusion chromatography (Superdex 200 increase 10/300 GL GE Healthcare) that had been pre-equilibrated in 50 mM Tris, 100 mM NaCl, pH 7.4 buffer in an AKTA FPLC system (GE Healthcare) for further purification to ensure the purity >95%.

### Stepwise polyprotein preparation with controlled sequences

Here we used the sample preparation for ubiquitin homo-polymer, Coh-tev-L-(Ub)_n_-NGL, as an example. Ligation unit Coh-tev-L-Ub-NGL was first linked to the GL-ELP_50nm_-C functionalized coverslip by OaAEP1 and the C-terminus was blocked. Then, the TEV protease solution was added on the coverslip to cleave the TEV site in the protein unit (0.4 mg mL^−1^ TEV protease 100 μL, 75 mM NaCl, 0.5 mM EDTA, 25 mM Tris-HCl, pH 8.0, 10% [v/v] glycerol). Typically, it was reacted for ~1 hour at 25 °C to produce GL-Ub-NGL-glass with exposed N-terminal GL residues. TEV protease was then washed away. Then, ~5 times the amount of the ligation unit, compared with the first time, was added to the solution for the stepwise ligation by OaAEP1. Due to the incomplete ligation reaction, we estimated that the real ratio between the two reactants was beyond 10. As a result, ubiquitin dimer was obtained on the glass surface as Coh-tev-L-(Ub)_2_-NGL-Glass. To reach the desired polymerization number N, this stepwise ligation and cleavage procedure was then repeated for N-1 times, leading to the protein polymer GL-(Ub)_n_-NGL-Glass. The final TEV cleavage was omitted to obtain Coh-tev-L-(Ub)_n_-NGL-Glass, which was ready for single-molecule AFM experiment using a Coh-XDoc system. A similar procedure was used to build other protein homo-polymer (Rd)_n_, and the hetero-polymer (Ub-Rd)_n_.

### Functionalized coverslip surface preparation

A glass coverslip (Sail Brand, China) surface was cleaned and activated by chromic acid treatment for 30 min at 80 °C. For amino-silanization, the coverslips were completely submerged in 1% (v/v) APTES toluene solution for 1 hour at room temperature, protected from light. The coverslips were then washed with toluene and absolute ethyl alcohol and dried under a stream of nitrogen. Then, the coverslips were incubated at 80 °C for 15 min. After immobilization, the coverslips were cooled down to room temperature. Two hundred microliters of sulfo-SMCC (1 mg mL^−1^) dimethyl sulfoxide (DMSO) solution was added between two immobilized coverslips and incubated for 1 h protected from light. The coverslips were washed with DMSO first and then with absolute ethyl alcohol to remove residual sulfo-SMCC. The cleaned coverslips were dried under a stream of nitrogen. 200 μL of 200 μM GL-ELP_50nm_-C protein solution was pipetted over a functional coverslip and was incubated for ~3 h. Finally, the coverslip was washed with Milli-Q water to remove the unreacted GL-ELP_50nm_-C and was used immediately or stored at 4 °C.

Cohesin-NGL was linked to the POIs, such as GL-(Ub)_n_-NGL if necessary. OaAEP1-catalyzed coupling of the bound GL-ELP_50nm_-C and Coh-POIs-NGL was done in the measurement buffer for 20–30 min. The sample cell was used for AFM-SMFS measurement after washing with the measurement buffer.

### Functionalized cantilevers surface preparation

Silicon nitride cantilever (MLCT, Bruker Corp) was used as a force probe. The surface chemistry of the cantilevers was similar to that of the coverslip. Cantilevers were cleaned by chromic acid treatment for 10 min at 80 °C. Cleaned cantilevers were functionalized by amino-silanization with APTES and were then conjugated to sulfo-SMCC. C-ELP_50nm_-NGL was linked to the surface with maleimide group of sulfo-SMCC for 1.5 h and the unreacted ELP was removed by Milli-Q water. The functionalized cantilever with ELP was immersed in 200 μL of 50 μM GL-CBM-XDoc protein solution containing 200 nM OaAEP1.

### Covalent attachment method

First, the silanized glass coverslip was functionalized with GL-ELP-C using thiol-maleimide chemistry in which the ELP was used as a spacer. Next, the polyprotein was covalently linked to the coverslip by OaAEP1 (Fig. 3a. Step 1) followed by ligation with Coh-NGL as Coh-(POI)_n_-glass (Step 2). Similarly, the C-ELP-NGL functionalized AFM tip was linked with GL-CBM-XDoc (Step 3). Consequently, the cohesin-dockerin pair formed when the tip pressed the coverslip as Tip-CBM-(XDoc-Coh)-(POI)_n_-glass (Step 4).

### AFM measurements

AFM-based SMFS experiments were performed on Nanowizard 4 AFM (JPK Germany). MLCT cantilever with a spring constant (*k*) of ~40 pN nm^−1^ was used. The equipartition theorem was used to calibrate the *k* of each cantilever in solution with an accurate value before the experiment. All proteins were measured in AFM measurement buffer (100 mM Tris, 100 mM NaCl, pH 7.4). For measurements using non-specific interaction, 10 μL of the polyprotein sample at a concentration of ~1 mg mL^−1^ was diluted into 30 μL of measurement buffer and added to a clean glass coverslip. The protein was allowed to absorb for 30 min. The suspending protein was washed away with 2 mL of the measurement buffer, and 1.5 mL measurement buffer was used to cover the coverslip. The tip of the AFM cantilever pressed the protein-deposited surface under a contact force of ~0.5 nN for hundreds of milliseconds, and a single polyprotein molecule was picked up with a ratio of ~0.01% and stretched at a constant pulling velocity of 400 nm s^−1^ in all experiments. The spring constant of the cantilevers used for (Ub)_n_ were of ~44 pN nm^−1^ (non-specific protein immobilization), and 45 pN nm^−1^ (specific immobilization), for Coh-(Ub)_6_ was of 39 pN nm^−1^, for (GB1-Fe(III)-Rd)_n_ were of ~56 pN nm^−1^ and ~48 pN nm^−1^, for (GB1-Zn-Rd)_n_ were of ~53 pN nm^−1^ and ~41 pN nm^−1^, for (Rd)_6_ was of ~34 pN nm^−1^, for (Ub)_6_ was of ~61 pN nm^−1^, for (Ub-Rd)_3_ was of ~100 pN nm^−1^, for (Ub-Rd)_5_ was of ~35 pN nm^−1^.

The data analysis of the force-extension curve was carried out using program Igor Pro 6.12 (Wavemetrics). The curves were fitted with the worm-like-chain (WLC) model of polymer elasticity, and the persistence length is of ~0.4 nm. For measurements with both covalent attachment and specific interaction pairs configuration, the functionalized glass coverslips and cantilevers were used. For the experiments using the functional coverslip and cantilever, only the curves containing the whole information of the protein construct and a high rupture force from the cohesin-dockerin dissociation were selected with a ratio of ~5%.

### Reporting summary

Further information on research design is available in the Nature Research Reporting Summary linked to this article.

## Supplementary information

Supplementary Information

Reporting Summary

Source Data

## Data Availability

Data supporting the findings of this work are available within the paper and its Supplementary Information files. A reporting summary for this Article is available as a Supplementary Information file. The datasets generated and analyzed during the current study are available from the corresponding author upon request. The source data underlying Figs. 2c, 3e, 4c, e, f are provided as a Source Data file.
