# Supplementary material for: Enzymatic biosynthesis and immobilization of polyprotein verified at the single-molecule level
Source: Nat Commun. 2019 Jun 24;10:2775. doi: 10.1038/s41467-019-10696-x (PMC6591319; doi:10.1038/s41467-019-10696-x)
Supplement: Supplementary file 1 — Supplementary Information [file 41467_2019_10696_MOESM1_ESM.pdf]

Enzymatic biosynthesis and immobilization of polyprotein verified at the single-molecule level

Deng *et al.*

Supplementary Methods

Protein engineering

First, the pQE80L-POI or pET28a-POI expression plasmids were transformed into *E. coli* BL21(DE3) cells. Single colonies was picked into LB medium containing 100 μg/mL ampicillin sodium salt or 50 μg/mL kanamycin (continuous shaking, 37°C, and 16-20 hours). After grown to saturation, overnight cultures were diluted 1:50 into fresh LB media containing ampicillin sodium salt or kanamycin (continuous shaking, 37°C, t ~3 hours. They were induced with 1 mM isopropyl β-D-thiogalactoside (IPTG) based on each protein when OD_{600} is ~ 0.6. The bacterial cultures were allowed to incubate for an additional 4-6 hours (37°C). Finally, 400 mL bacterial culture was pelleted by centrifugation (13260 g, 25 min, 4°C) and stored at -80°C before purification.

The cells were then resuspended in 25mL lysis buffer 50 mM Tris, pH7.4) and lysed on ice using a Biosafer sonicator (15% amplitude for 30 min). The lysate was centrifuged (19632 g, 40 min, 4°C) to pellet cell fragments and the supernatant fluids were applied to a Co-NTA or Ni-NTA affinity column (TALON) and washed with buffer containing 20 mM Tris, 400 mM NaCl, 2 mM Imidazole, pH 7.4. The bound protein was eluted with high-concentration imidazole buffer (20 mM Tris, 400 mM NaCl, 250 mM imidazole, pH 7.4). For rubredoxin, we firstly used an anion exchange chromatography (Mono Q 5/50 GL GE Healthcare) applying a continuous salt gradient of 0–30% of buffer B (50 mM Tris, 1 M NaCl, pH 8.5) to separate iron and zinc form. Then a size-exclusion chromatography (Superdex 200 increase 10/300 GL GE Healthcare) that had been pre-equilibrated in 50 mM Tris, 100 mM NaCl, pH 7.4 buffer in an AKTA FPLC system (GE Healthcare) was used for further purification to ensure the purity >95%.

TEV protease cleaves Histag-tev-L-POI-NGL protein at the TEV cleavage site "ENLYFQ/G". This proteolysis removed cohesin or Histag in the buffer containing 75 mM NaCl, 0.5 mM EDTA, 25 mM Tris-HCl, pH 8.0, 10% [v/v] glycerol at 25°C for ~1.5 hours. After the reaction, the TEV protease-protein reaction mixture was loaded to a Co-NTA affinity column, and the unbound solution was collected.

Two different lengths of ELPs with cysteines were used in this study. ELP_{50nm} consists of 24 repeats of VPGXG with a contour length of 54.7 nm, and ELP_{40nm} consists of 16 repeats with a contour length of 37.8 nm. As the cysteine concentration was equivalent to proteins of ELPs, ELPs concentration was confirmed with the Ellman method, in which 10 μL of protein incubated with 20 μL of DTNB solution (0.5 mM 5,5'-dithiobis(2-nitrobenzoic acid), 99%, Alfa Aesar), 1 mM EDTA, 7.2 M Urea) for 30 min at room temperature. The absorbance of protein and DTNB reaction solution was measured at 412 nm (ε = 13700 M^{-1}) by Nanodrop 2000 spectrophotometer. A mixture containing 10 μL of elution buffer and 20 μL of DTNB solution was used as a blank.

OaAEP1 was activated at pH 3.7. Typically, 0.2-2 μM activated OaAEP1 was added to the target protein solution at Tris buffer (100 mM Tris, 100 mM NaCl, pH 7.4) and incubated for ~ 30 min at room temperature (above 25°C) or 37°C. After incubation, the degree of polymerization was analyzed by SDS-PAGE gel, as shown in Figure 1. Moreover, size-exclusion analytical chromatography (Superdex 75 Increase 10/300 GL column) using an AKTA FPLC system at a flow rate of 0.3 mL min^{-1} was used to quantitatively obtain the polymerization degree.

To test the pH dependence of OaAEP1 reaction, the pH of 400 μM GL-Ub-NGL protein solution in the succinate-phosphate-glycine buffer (12.5 mM succinic acid, 43.75 mM NaH_2PO_4, 43.75 mM glycine) was adjusted pH from 3 to 8 using 1 mM NaOH solution. After added 8 μM OaAEP1, the solution was incubated at 30℃ for 30 minutes.

OaAEP1 ligation efficiency test

To check the ratio dependency of ligation efficiency between the two reactants, solutions of Coh-tev-L-Ub-NGL and GL-Ub were mixed with the addition of 0.1 μM OaAEP1 at 30℃ for 30 minutes. The solution with a 1:1 ratio between two reactants contained 5 μM Coh-tev-L-Ub-NGL and 5 μM GL-Ub. Then, 15 μM, 25 μM, and 50 μM of Coh-tev-L-Ub-NGL were tested, respectively (Figure 5). To calculate the efficiency based on the much smaller reactant monomer GL-Ub, ten times the concentration of each reactant was added. (Supplementary Fig.3). The ligation was stopped in SDS loading buffer and characterized by SDS-PAGE gel. The ligation efficiency for each reaction was calculated based on the band intensity of the product/reactant using the software image J. The ratio of TEV Protease cleavage was tested by mixing 20 μM Coh-tev-L-Ub and 2 μM TEV Protease and incubating the solution at 25℃for 2 h.

In order to prove the exposed GL-POI after TEV Protease cleavage could be linked to the NGL-POI by OaAEP1, we used TEV Protease to cut Coh-tev-L-Ub and loaded the solution to a Ni-NTA affinity column. The unbound solution, which contained the GL-Ub without His-tag, was collected and concentrated. Solutions of 5 μM Coh-tev-L-Ub-NGL, 5 μM GL-Ub and 0.1 μM OaAEP1 were mixed and incubated at 30℃ for 30 minutes. The reaction was stopped using SDS loading buffer and characterized by SDS-PAGE gel directly.

Supplementary Notes

Protein sequences

GL-Ub-**NGL**

GLHHHHHHGSMQIFVKTLTGKTITLEVEPSDTIENVKAKIQDKEGIPPDQQRLIFA
GKQLEDGRTLSDYNIQKESTLHLVLRLRAARS**NGL**

GL-Rd-**NGL**

GLHHHHHHGSMKKYTCTVCGYIYNPEDGDPDNGVNPGTDFKDIPDDWVCPLCG
VGKDQFEEVEERSVPGVG**NGL**

Coh-**NGL**

RGSHHHHHHGSMGTALTDRGMTYDLDPKDGSSAATKPVLEVTKKVFDTAADAA
GQTVTVEFKVSGAEGKYATTGYHIYWDERLEVVATKTGAYAKKGAALEDSSLAK
AENNGNGVFVASGADDDFGADGVMWTVELKVPADAKAGDVYPIDVAYQWDPS
KGDLFTDNKDSAQGKLMQAYFFTQGIKSSSNPSTDEYLVKANATYADGYIAIKAG
EPRS**NGL**

GL-CBM-Xmodule-Dockerin (GL-CBM-XDoc)

GLHHHHHHGSGMANTPVSGNLKVEFYNSNPSDTTNSINPQFKVTNTGSSAIDLS
KLTLRYYYTVDGQKDQTFWSDHAAIIGSNGSYNGITSNVKGTFVKMSSSTNNAD
TYLEISFTGGTLEPGAHVQIQGRFAKNDWSNYTQSNDYSFKSASQFVEWDQVTA
YLNGVLVWGKEPGTSGSVVPSTQPVTTPPATTKPPATTIPPSDDPNAVVPTSGGSRS
GGNTVTSAVKTQYVEIESVDGFYFNTEDKFDTAQIKKAVLHTVYNEGYTGDDGV
AVVLREYESEPVDITAELTFGDATPANTYKAVENKFDYEIPVYYNNATLKDAEGN
DATVTVYIGLKGDTDLNNIVDGRDATATLTYYAATSTDGKDATTVALSPSTLVGG
NPESVYDDFSAFLSDVKVDAGKELTRFAKKAERLIDGRDASSILTFYTKSSVDQY

KDMAANEPNKLWDIVTGDARS

GL-Ub

GLHHHHHHGSMQIFVKTLTGKTITLEVEPSDTIENVKAKIQDKEGIPPDQQRLIFA

GKQLEDGRTLSDYNIQKESTLHLVLRLRAARSLPETGG

Coh-**tev**-**L**-Ub

HHHHHHGSMGTALTDRGMTYDLDPKDGSSAATKPVLEVTKKVFDTAADAAGQT

VTVEFKVSGAEGKYATTGYHIYWDERLEVVATKTGAYAKKGAALEDSSLAKAEN

NGNGVFVASGADDDFGADGVMWTVELKVPADAKAGDVYPIDVAYQWDPSKGD

LFTDNKDSAQGKLMQAYFFTQGIKSSSNPSTDEYLVKANATYADGYIAIKAGEPR

S**ENLYFQGL**GRSMQIFVKTLTGKTITLEVEPSDTIENVKAKIQDKEGIPPDQQRLIF

AGKQLEDGRTLSDYNIQKESTLHLVLRLRAARSLPETGG

GL-GB1-*cp*Rd-**NGL**
GLHHHHHHGSRSGSMDTYKLILNGKTLKGETTTEAVDAATAEKVFKQYANDNG
VDGEWTYDDATKTFTVTERSMKKYTCTVCGYIYNPEDGDPDNGVNPGTDFKDIP
DDWVCPLCGVGKDQFEEVEERSVPGVG**NGL**

G -GB1-*pf*Rd-**NGL**
GLHHHHHHGSRSGSMDTYKLILNGKTLKGETTTEAVDAATAEKVFKQYANDNG
VDGEWTYDDATKTFTVTERSAKWVCKICGYIYDEDAGDPDNGISPGTKFEELPD
DWVCPICGAPKSEFEKLEDRSVPGVG**NGL**

Coh-**tev**-**L**-Rd-**NGL**

HHHHHHGSMGTALTDRGMTYDLDPKDGSSAATKPVLEVTKKVFDTAADAAGQT

VTVEFKVSGAEGKYATTGYHIYWDERLEVVATKTGAYAKKGAALEDSSLAKAEN

NGNGVFVASGADDDFGADGVMWTVELKVPADAKAGDVYPIDVAYQWDPSKGD

LFTDNKDSAQGKLMQAYFFTQGIKSSSNPSTDEYLVKANATYADGYIAIKAGEPR

SEF**ENLYFQGL**GSMKKYTCTVCGYIYNPEDGDPDNGVNPGTDFKDIPDDWVCPL

CGVGKDQFEEVEERSVPGVG**NGL**

Coh-**tev-L**-Ub-**NGL**

HHHHHHGSMGTALTDRGMTYDLDPKDGSSAATKPVLEVTKKVFDTAADAAGQT

VTVEFKVSGAEGKYATTGYHIYWDERLEVVATKTGAYAKKGAALEDSSLAKAEN

NGNGVFVASGADDDFGADGVMWTVELKVPADAKAGDVYPIDVAYQWDPSKGD

LFTDNKDSAQGKLMQAYFFTQGIKSSSNPSTDEYLVKANATYADGYIAIKAGEPR

S**ENLYFQGL**GRSMQIFVKTLTGKTITLEVEPSDTIENVKAKIQDKEGIPPDQQRLIF

AGKQLEDGRTLSDYNIQKESTLHLVLRLRAARS**NGL**

GL-ELP$_{50nm}$-C

GLHHHHHHGSVPGVGVPGVGVPGEGVPGVGVPGVGVPGVGVPGVGVPGEGVP

GGLRSVPGVGVPGVGVPGEGVPGVGVPGVGVPGVGVPGVGVPGEGVPGGLRSV

PGVGVPGVGVPGEGVPGVGVPGVGVPGVGVPGVGVPGEGVPGGLRS**C**

ELP$_{50nm}$-C

RGSHHHHHHGSVPGVGVPGVGVPGEGVPGVGVPGVGVPGVGVPGVGVPGEGV

PGGLRSVPGVGVPGVGVPGEGVPGVGVPGVGVPGVGVPGVGVPGEGVPGGLRS

VPGVGVPGVGVPGEGVPGVGVPGVGVPGVGVPGVGVPGEGVPGGLRS**C**

C-ELP$_{40nm}$-**NGL**

RGSHHHHHHGS**C**VPGGLRSVPGVGVPGVGVPGEGVPGVGVPGVGVPGVGVPG

VGVPGEGVPGGLRSVPGVGVPGVGVPGEGVPGVGVPGVGVPGVGVPGVGVPGE

GVPGGLRS**NGL**

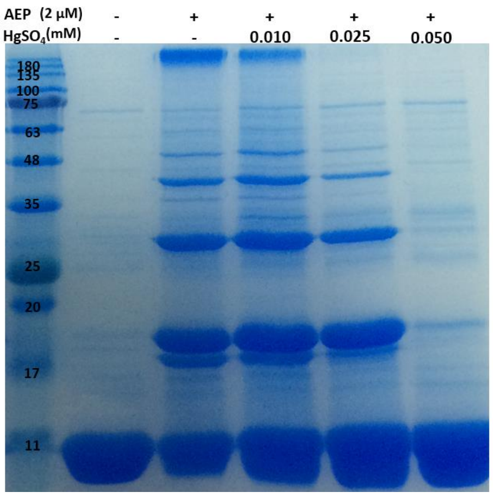

Supplementary Figure 1. The SDS-PAGE gel results of OaAEP1-mediate ubiquitin coupling under toxic mercury ion. It indicated that the coupling reaction was not affected by Hg when the concentration was below 0.025 mM.

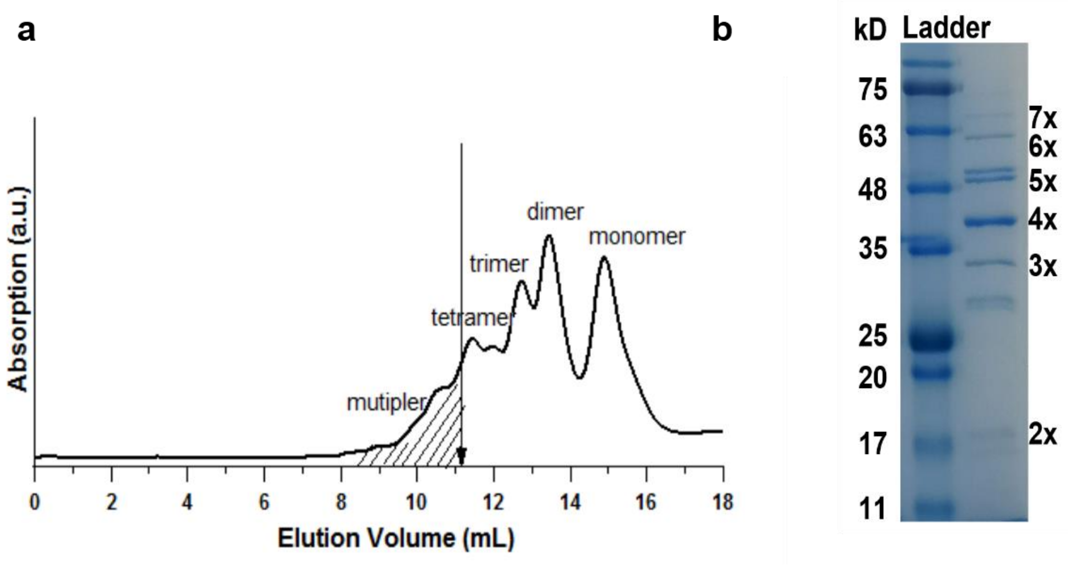

Supplementary Figure 2. The preparation of $(Ub)_n$ sample for single-molecule AFM experiments. **a** The gel-filtration profile performed by FPLC to eliminate unreacted Ub monomer and dimer for better AFM performance. **b** The SDS-PAGE gel result of the gel-filtration purified $(Ub)_n$ sample shows that the tetramer presents most after the purification.

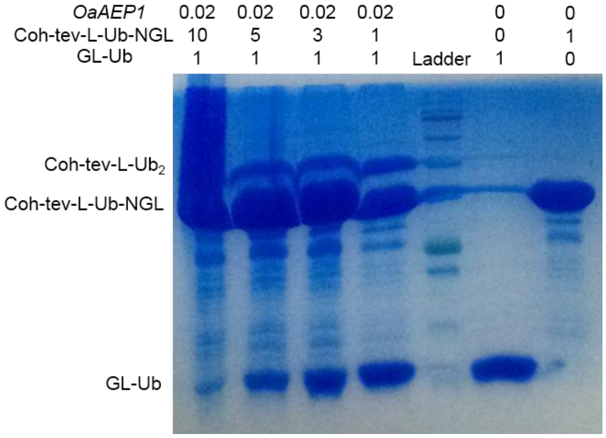

Supplementary Figure 3. SDS-PAGE gel results showed the OaAEP1 ligation efficiency is proportional to the ratio between the two reactants. The yield is calculated based on the decrease of the band intensity of the monomer GL-Ub, using the software image J. Here, ten times the concentration of each reactant is added compared with the reactant for Figure 5, for clear band detection of the small monomer GL-Ub. The efficiency increases from 20% when the ratio is 1 to 1, to 40% when the ratio is 3 to 1, to 50% when the ratio is 5 to 1 and to 85% when the ratio is 10 to 1.

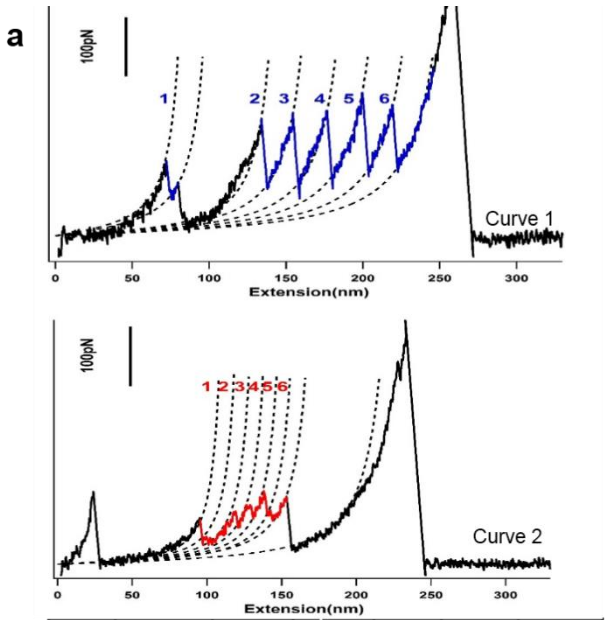

b

peak	1	2	3	4	5	6
Ub (6)						
# (%)	6 (2%)	67 (19%)	96 (28%)	84 (24%)	48 (14%)	45(13%)
Rd (6)						
# (%)	60 (16%)	91 (24%)	76 (20%)	74 (19%)	45(12%)	35(9%)

Supplementary Figure 4. AFM results for homo-polyprotein $(Ub)_6$, and $(Rd)_6$. **a** Representative curves with maximum unfolding peaks from the unfolding experiments of homo-polyprotein $(Ub)_6$, (curve 1), and $(Rd)_6$, (curve 2). **b** The table shows the statistical analysis of the curve number with specific unfolding peaks for $(Ub)_6$ and $(Rd)_6$.

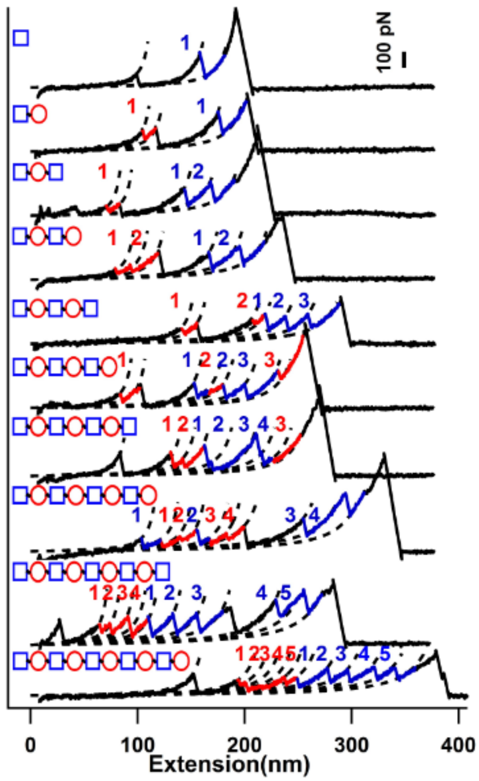

Supplementary Figure 5. Representative force-extension curves with maximum unfolding peaks from the unfolding experiments of hetero-polyprotein (Ub-Rd)$_5$, prepared from the stepwise polymerization procedure. Blue square represents Ub and red circle represents Rd. Similarly, the unfolding peaks from Ub are colored in blue and unfolding peaks from Rd are colored in red.
